# Tetra­bromido­[4-(triphenyl­phosphanyl­oxy)but­yl]tellurium acetonitrile monosolvate

**DOI:** 10.1107/S1600536812051707

**Published:** 2013-01-09

**Authors:** Sari M. Närhi, Raija Oilunkaniemi, Risto S. Laitinen

**Affiliations:** a Department of Chemistry, PO Box 3000, FI-90014 University of Oulu, Finland

## Abstract

In the title compound, [TeBr_4_(C_22_H_23_OP)]·CH_3_CN, the Te atom exhibits a square-pyramidal coordination with an apical Te—C bond and four basal Te—Br bonds. The conformation of the aliphatic C—C—C—C chain is *gauche* [torsion angle = −67.7 (8)°]. A weak C—H⋯Br inter­action helps to establish the conformation. In the crystal, there is a weak secondary bonding inter­action [Te⋯N = 3.456 (11) Å] between the Te atom and the N atom of the solvent mol­ecule, which completes a distorted TeNCBr_4_ octa­hedron. Inversion dimers linked by pairs of C—H⋯Br inter­actions are also observed.

## Related literature

For the formation of Ph_3_PO(CH_2_)_4_TeBr_4_ and the structure of the dichloro­methane monosolvate, see: Kunnari *et al.* (2001 ▶). For Te⋯N inter­actions, see: Cozzolino *et al.* (2011 ▶); Pauling (1960 ▶).
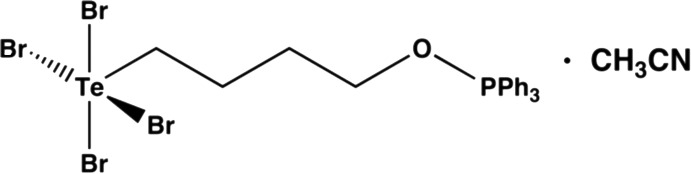


## Experimental

### 

#### Crystal data

[TeBr_4_(C_22_H_23_OP)]·C_2_H_3_N
*M* *_r_* = 822.67
Monoclinic, 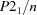

*a* = 9.3195 (19) Å
*b* = 13.899 (3) Å
*c* = 21.962 (4) Å
β = 94.92 (3)°
*V* = 2834.3 (10) Å^3^
*Z* = 4
Mo *K*α radiation
μ = 6.76 mm^−1^
*T* = 150 K
0.10 × 0.10 × 0.05 mm

#### Data collection

Bruker–Nonius KappaCCD diffractometer
Absorption correction: multi-scan (*SADABS*; Sheldrick, 2008 ▶) *T*_min_ = 0.551, *T*_max_ = 0.729
11187 measured reflections
4684 independent reflections
3714 reflections with *I* > 2σ(*I*)
*R*_int_ = 0.073

#### Refinement

*R*[*F* ^2^ > 2σ(*F* ^2^)] = 0.048
*wR*(*F* ^2^) = 0.135
*S* = 1.07
4684 reflections
291 parameters
H-atom parameters constrained
Δρ_max_ = 0.88 e Å^−3^
Δρ_min_ = −0.79 e Å^−3^

### 

Data collection: *COLLECT* (Bruker, 2008 ▶); cell refinement: *DENZO-SMN* (Otwinowski & Minor, 1997 ▶); data reduction: *DENZO-SMN*; program(s) used to solve structure: *SHELXS97* (Sheldrick, 2008 ▶); program(s) used to refine structure: *SHELXL97* (Sheldrick, 2008 ▶); molecular graphics: *DIAMOND* (Brandenburg, 2006) ▶; software used to prepare material for publication: *WinGX* (Farrugia, 2012 ▶).

## Supplementary Material

Crystal structure: contains datablock(s) I, global. DOI: 10.1107/S1600536812051707/hb7012sup1.cif

Structure factors: contains datablock(s) I. DOI: 10.1107/S1600536812051707/hb7012Isup2.hkl

Additional supplementary materials:  crystallographic information; 3D view; checkCIF report

## Figures and Tables

**Table 1:** Selected bond lengths (Å)

Te1—C4	2.176 (7)
Te1—Br2	2.6652 (10)
Te1—Br4	2.6814 (11)
Te1—Br1	2.6944 (11)
Te1—Br3	2.7201 (10)

**Table 2:** Hydrogen-bond geometry (Å, °)

*D*—H⋯*A*	*D*—H	H⋯*A*	*D*⋯*A*	*D*—H⋯*A*
C3—H3*B*⋯Br1	0.99	2.82	3.450 (7)	122
C26—H26⋯Br3^i^	0.95	2.75	3.619 (8)	152

Symmetry code: (i) 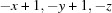.

**Fig. 1.**
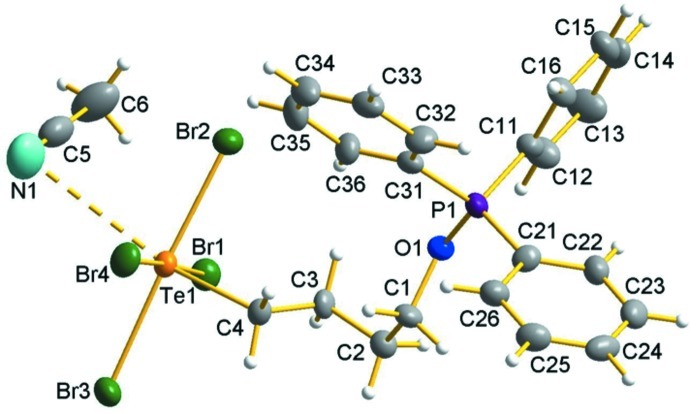
The molecular structure with displacement ellipsoids drawn at 50% probability.

**Fig. 2.**
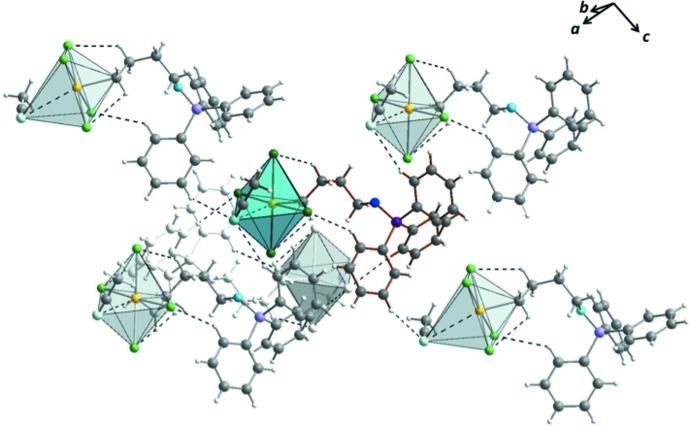
The packing of the zwitterions and the solvent indicating the short inter- and intramolecular contacts < 3.00 Å.

## supplementary crystallographic information

### Comment

The formation of Ph_3_PO(CH_2_)_4_TeBr_4_ has been reported earlier by us (Kunnari *et al.* 2001). The formally zwitterionic compound was isolated by treating TeBr_4_ with an equimolar amount of triphenylphosphine in tetrahydrofuran. The compound was crystallized from dichloromethane and its molecular structure was determined as a dichloromethane solvate. The title compound was recrystallized from acetonitrile and consequently contains acetonitrile solvent molecules. It is isomorphic with the CH_2_Cl_2_ solvate.

The molecular structure of the title compound indicating the numbering of the atoms is shown in Fig. 1. The Te—Br bond lengths range from 2.6652 (10) to 2.7201 (10) Å. The Te—C bond length is 2.176 (7) Å and the P—O bond length is 1.568 (5) Å. These can be compared to the bond lengths in the corresponding CH_2_Cl_2_ solvate in which the Te—Br bond lengths range 2.6776 (8) - 2.6952 (9) Å, Te—C bond length is 2.177 (6) Å, and P—O bond length is 1.581 (4) Å (Kunnari *et al.* 2001).

The closest internuclear contact from tellurium atom to the nitrogen atom of the solvent molecule is 3.456 (11) Å expanding the square pyramidal coordination polyhedron into a distorted octahedron. This is typical for Te^···^N secondary bonding interactions (Cozzolino *et al.* 2011). Interestingly, the Te^..^N interaction is stronger [Pauling (1960) bond order is 0.15] compared to the Te^..^Cl interaction in the CH_2_Cl_2_ solvate (Kunnari *et al.* 2001), for which the contact is 4.175 (3) Å corresponding to the Pauling bond order of only 0.09. This is also reflected in the C—Te—E (E = N, Cl) bond angles which are 165.0 (3) and 144.0 (1) °, respectively.

Intra- and intermolecular hydrogen bonds link the zwitterions into a three- dimensional network, as shown in Fig. 2. The shortest intermolecular C—H^···^Br contacts span a range of 2.75 (1)–2.96 (1) Å and the short C—H^..^N contacts are 2.68 (1) and 2.96 (1) Å.

### Experimental

Yellow plates of the title compound were obtained from the acetonitrile solution of Ph_3_PO(CH_2_)_4_TeBr_4_ by slow evaporation of the solvent.

### Refinement

H atoms were positioned geometrically and refined using a riding model with C—H = 0.99 Å and with *U*_iso_(H) = 1.2 *U*_eq_(C), 0.98 Å and *U*_iso_(H) = 1.5 *U*_eq_(C) and 0.95 Å and *U*_iso_(H) = 1.2 *U*_eq_(C)for the methylene, methyl and aromatic H atoms, respectively.

### Crystal data

**Table:**

[TeBr_4_(C_22_H_23_OP)]·C_2_H_3_N	*F*(000) = 1568
*M**_r_* = 822.67	*D*_x_ = 1.928 Mg m^−^^3^
Monoclinic, *P*2_1_/*n*	Mo *K*α radiation, λ = 0.71073 Å
Hall symbol: -P 2yn	Cell parameters from 3714 reflections
*a* = 9.3195 (19) Å	θ = 2.3–25.0°
*b* = 13.899 (3) Å	µ = 6.76 mm^−^^1^
*c* = 21.962 (4) Å	*T* = 150 K
β = 94.92 (3)°	Plate, yellow
*V* = 2834.3 (10) Å^3^	0.10 × 0.10 × 0.05 mm
*Z* = 4	

### Data collection

**Table:**

Bruker–Nonius KappaCCD diffractometer	4684 independent reflections
Radiation source: fine-focus sealed tube	3714 reflections with *I* > 2σ(*I*)
Graphite monochromator	*R*_int_ = 0.073
φ scans, and ω scans with κ offsets	θ_max_ = 25.0°, θ_min_ = 2.3°
Absorption correction: multi-scan (*SADABS*; Sheldrick, 2008)	*h* = −11→10
*T*_min_ = 0.551, *T*_max_ = 0.729	*k* = −15→16
11187 measured reflections	*l* = −24→26

### Refinement

**Table:**

Refinement on *F*^2^	Secondary atom site location: difference Fourier map
Least-squares matrix: full	Hydrogen site location: inferred from neighbouring sites
*R*[*F*^2^ > 2σ(*F*^2^)] = 0.048	H-atom parameters constrained
*wR*(*F*^2^) = 0.135	*w* = 1/[σ^2^(*F*_o_^2^) + (0.0606*P*)^2^ + 8.1058*P*] where *P* = (*F*_o_^2^ + 2*F*_c_^2^)/3
*S* = 1.07	(Δ/σ)_max_ < 0.001
4684 reflections	Δρ_max_ = 0.88 e Å^−^^3^
291 parameters	Δρ_min_ = −0.79 e Å^−^^3^
0 restraints	Extinction correction: *SHELXS97* (Sheldrick, 2008), Fc^*^=kFc[1+0.001xFc^2^λ^3^/sin(2θ)]^-1/4^
Primary atom site location: structure-invariant direct methods	Extinction coefficient: 0.0024 (3)

### Special details

**Table:**

Geometry. All e.s.d.'s (except the e.s.d. in the dihedral angle between two l.s. planes) are estimated using the full covariance matrix. The cell e.s.d.'s are taken into account individually in the estimation of e.s.d.'s in distances, angles and torsion angles; correlations between e.s.d.'s in cell parameters are only used when they are defined by crystal symmetry. An approximate (isotropic) treatment of cell e.s.d.'s is used for estimating e.s.d.'s involving l.s. planes.
Refinement. Refinement of *F*^2^ against ALL reflections. The weighted *R*-factor *wR* and goodness of fit *S* are based on *F*^2^, conventional *R*-factors *R* are based on *F*, with *F* set to zero for negative *F*^2^. The threshold expression of *F*^2^ > σ(*F*^2^) is used only for calculating *R*-factors(gt) *etc*. and is not relevant to the choice of reflections for refinement. *R*-factors based on *F*^2^ are statistically about twice as large as those based on *F*, and *R*- factors based on ALL data will be even larger.

### Fractional atomic coordinates and isotropic or equivalent isotropic displacement parameters (Å^2^)

**Table:**

	*x*	*y*	*z*	*U*_iso_*/*U*_eq_	
Te1	0.55868 (5)	0.29462 (3)	−0.09855 (2)	0.02466 (19)	
Br1	0.40006 (9)	0.21866 (6)	−0.19512 (3)	0.0335 (2)	
Br2	0.50893 (9)	0.14024 (6)	−0.03152 (4)	0.0356 (2)	
Br3	0.59404 (9)	0.45190 (6)	−0.16944 (4)	0.0388 (2)	
Br4	0.71342 (9)	0.37800 (6)	−0.00436 (4)	0.0380 (2)	
P1	0.0720 (2)	0.26347 (14)	0.10968 (8)	0.0248 (4)	
O1	0.0848 (5)	0.2852 (3)	0.0403 (2)	0.0275 (11)	
C1	0.1193 (8)	0.3822 (5)	0.0189 (3)	0.0288 (17)	
H1A	0.2167	0.4017	0.0361	0.035*	
H1B	0.0488	0.4295	0.0321	0.035*	
C2	0.1130 (8)	0.3783 (6)	−0.0504 (3)	0.0303 (17)	
H2A	0.1202	0.4447	−0.0660	0.036*	
H2B	0.0177	0.3527	−0.0660	0.036*	
C3	0.2297 (7)	0.3172 (5)	−0.0764 (3)	0.0269 (16)	
H3A	0.2302	0.2520	−0.0582	0.032*	
H3B	0.2090	0.3108	−0.1212	0.032*	
C4	0.3740 (8)	0.3632 (5)	−0.0625 (3)	0.0285 (17)	
H4A	0.3930	0.3671	−0.0175	0.034*	
H4B	0.3680	0.4300	−0.0781	0.034*	
C11	−0.0254 (8)	0.1540 (5)	0.1093 (3)	0.0281 (16)	
C12	−0.0930 (9)	0.1166 (6)	0.0554 (4)	0.041 (2)	
H12	−0.0853	0.1485	0.0176	0.049*	
C13	−0.1719 (10)	0.0320 (6)	0.0577 (4)	0.049 (2)	
H13	−0.2202	0.0065	0.0214	0.059*	
C14	−0.1801 (9)	−0.0147 (6)	0.1125 (4)	0.042 (2)	
H14	−0.2315	−0.0737	0.1134	0.050*	
C15	−0.1146 (9)	0.0226 (6)	0.1663 (4)	0.0385 (19)	
H15	−0.1236	−0.0098	0.2039	0.046*	
C16	−0.0364 (9)	0.1066 (5)	0.1656 (3)	0.0329 (18)	
H16	0.0093	0.1323	0.2024	0.039*	
C21	−0.0209 (8)	0.3556 (5)	0.1456 (3)	0.0284 (17)	
C22	−0.1690 (8)	0.3508 (5)	0.1482 (3)	0.0304 (17)	
H22	−0.2204	0.2960	0.1323	0.036*	
C23	−0.2421 (9)	0.4246 (6)	0.1736 (3)	0.0371 (19)	
H23	−0.3432	0.4202	0.1758	0.045*	
C24	−0.1682 (9)	0.5055 (6)	0.1959 (3)	0.0356 (19)	
H24	−0.2189	0.5570	0.2128	0.043*	
C25	−0.0214 (9)	0.5113 (6)	0.1936 (4)	0.039 (2)	
H25	0.0288	0.5665	0.2096	0.046*	
C26	0.0539 (9)	0.4379 (5)	0.1684 (3)	0.0340 (18)	
H26	0.1551	0.4428	0.1665	0.041*	
C31	0.2498 (8)	0.2506 (5)	0.1460 (3)	0.0294 (17)	
C32	0.2787 (9)	0.2633 (6)	0.2092 (3)	0.0364 (19)	
H32	0.2041	0.2809	0.2339	0.044*	
C33	0.4197 (9)	0.2496 (6)	0.2351 (4)	0.040 (2)	
H33	0.4415	0.2593	0.2777	0.048*	
C34	0.5280 (9)	0.2221 (6)	0.1991 (4)	0.042 (2)	
H34	0.6233	0.2132	0.2172	0.050*	
C35	0.4976 (9)	0.2076 (6)	0.1372 (4)	0.045 (2)	
H35	0.5709	0.1867	0.1128	0.054*	
C36	0.3595 (8)	0.2239 (6)	0.1110 (4)	0.0371 (19)	
H36	0.3397	0.2165	0.0681	0.044*	
N1	0.8684 (11)	0.1649 (8)	−0.1169 (5)	0.075 (3)	
C5	0.7977 (12)	0.1025 (8)	−0.1349 (5)	0.052 (2)	
C6	0.7060 (13)	0.0250 (8)	−0.1577 (6)	0.073 (3)	
H6A	0.6813	−0.0153	−0.1235	0.110*	
H6B	0.7565	−0.0139	−0.1864	0.110*	
H6C	0.6177	0.0515	−0.1788	0.110*	

### Atomic displacement parameters (Å^2^)

**Table:**

	*U*^11^	*U*^22^	*U*^33^	*U*^12^	*U*^13^	*U*^23^
Te1	0.0209 (3)	0.0284 (3)	0.0246 (3)	0.00017 (19)	0.00156 (19)	−0.00170 (19)
Br1	0.0357 (5)	0.0387 (5)	0.0257 (4)	−0.0025 (3)	0.0001 (3)	−0.0071 (3)
Br2	0.0376 (5)	0.0335 (5)	0.0356 (4)	0.0018 (3)	0.0013 (3)	0.0055 (3)
Br3	0.0325 (5)	0.0403 (5)	0.0441 (5)	−0.0040 (4)	0.0061 (3)	0.0102 (4)
Br4	0.0295 (5)	0.0459 (5)	0.0370 (4)	−0.0024 (4)	−0.0062 (3)	−0.0085 (4)
P1	0.0229 (10)	0.0277 (10)	0.0240 (9)	−0.0022 (8)	0.0038 (7)	0.0036 (8)
O1	0.029 (3)	0.027 (3)	0.026 (3)	0.000 (2)	0.001 (2)	0.004 (2)
C1	0.034 (4)	0.027 (4)	0.026 (4)	−0.001 (3)	0.003 (3)	0.007 (3)
C2	0.030 (4)	0.034 (4)	0.027 (4)	−0.002 (3)	0.002 (3)	0.002 (3)
C3	0.023 (4)	0.036 (4)	0.023 (4)	0.000 (3)	0.008 (3)	−0.002 (3)
C4	0.024 (4)	0.029 (4)	0.033 (4)	0.001 (3)	0.005 (3)	−0.001 (3)
C11	0.032 (4)	0.025 (4)	0.027 (4)	−0.001 (3)	0.003 (3)	0.002 (3)
C12	0.048 (5)	0.043 (5)	0.029 (4)	−0.010 (4)	−0.008 (4)	0.003 (4)
C13	0.055 (6)	0.044 (5)	0.046 (5)	−0.015 (4)	−0.017 (4)	0.000 (4)
C14	0.027 (5)	0.030 (4)	0.067 (6)	−0.005 (3)	0.001 (4)	−0.001 (4)
C15	0.043 (5)	0.032 (4)	0.043 (5)	−0.008 (4)	0.014 (4)	0.000 (4)
C16	0.040 (5)	0.034 (4)	0.026 (4)	−0.004 (4)	0.009 (3)	−0.001 (3)
C21	0.031 (4)	0.031 (4)	0.022 (4)	0.003 (3)	−0.002 (3)	−0.003 (3)
C22	0.026 (4)	0.036 (4)	0.029 (4)	−0.004 (3)	0.001 (3)	0.001 (3)
C23	0.037 (5)	0.043 (5)	0.033 (4)	0.004 (4)	0.012 (4)	0.008 (4)
C24	0.053 (6)	0.033 (4)	0.022 (4)	0.014 (4)	0.009 (4)	0.001 (3)
C25	0.047 (5)	0.031 (4)	0.036 (4)	−0.001 (4)	−0.002 (4)	−0.001 (4)
C26	0.039 (5)	0.029 (4)	0.035 (4)	0.002 (3)	0.007 (4)	0.003 (3)
C31	0.027 (4)	0.026 (4)	0.034 (4)	−0.003 (3)	−0.002 (3)	0.009 (3)
C32	0.040 (5)	0.039 (5)	0.029 (4)	0.003 (4)	−0.004 (3)	0.004 (4)
C33	0.036 (5)	0.036 (5)	0.045 (5)	−0.006 (4)	−0.008 (4)	0.013 (4)
C34	0.030 (5)	0.049 (5)	0.045 (5)	−0.003 (4)	−0.006 (4)	0.014 (4)
C35	0.028 (5)	0.058 (6)	0.051 (5)	−0.008 (4)	0.006 (4)	0.004 (4)
C36	0.024 (4)	0.047 (5)	0.040 (4)	−0.001 (4)	0.002 (3)	0.005 (4)
N1	0.071 (7)	0.074 (7)	0.084 (7)	0.010 (6)	0.031 (6)	−0.015 (6)
C5	0.054 (6)	0.050 (6)	0.054 (6)	0.021 (5)	0.015 (5)	0.006 (5)
C6	0.071 (8)	0.057 (7)	0.088 (8)	0.016 (6)	−0.016 (6)	−0.019 (6)

### Geometric parameters (Å, º)

**Table:**

Te1—C4	2.176 (7)	C15—H15	0.9500
Te1—Br2	2.6652 (10)	C16—H16	0.9500
Te1—Br4	2.6814 (11)	C21—C22	1.387 (11)
Te1—Br1	2.6944 (11)	C21—C26	1.410 (10)
Te1—Br3	2.7201 (10)	C22—C23	1.375 (11)
P1—O1	1.568 (5)	C22—H22	0.9500
P1—C21	1.769 (7)	C23—C24	1.386 (11)
P1—C11	1.772 (7)	C23—H23	0.9500
P1—C31	1.785 (8)	C24—C25	1.375 (12)
O1—C1	1.473 (8)	C24—H24	0.9500
C1—C2	1.518 (10)	C25—C26	1.380 (11)
C1—H1A	0.9900	C25—H25	0.9500
C1—H1B	0.9900	C26—H26	0.9500
C2—C3	1.529 (10)	C31—C36	1.382 (11)
C2—H2A	0.9900	C31—C32	1.403 (10)
C2—H2B	0.9900	C32—C33	1.399 (11)
C3—C4	1.497 (10)	C32—H32	0.9500
C3—H3A	0.9900	C33—C34	1.388 (12)
C3—H3B	0.9900	C33—H33	0.9500
C4—H4A	0.9900	C34—C35	1.380 (12)
C4—H4B	0.9900	C34—H34	0.9500
C11—C12	1.394 (10)	C35—C36	1.383 (11)
C11—C16	1.412 (10)	C35—H35	0.9500
C12—C13	1.390 (11)	C36—H36	0.9500
C12—H12	0.9500	N1—C5	1.140 (14)
C13—C14	1.375 (12)	C5—C6	1.438 (15)
C13—H13	0.9500	C6—H6A	0.9800
C14—C15	1.384 (12)	C6—H6B	0.9800
C14—H14	0.9500	C6—H6C	0.9800
C15—C16	1.378 (11)		
			
C4—Te1—Br2	88.4 (2)	C13—C14—H14	119.4
C4—Te1—Br4	85.4 (2)	C15—C14—H14	119.4
Br2—Te1—Br4	91.68 (3)	C16—C15—C14	120.2 (8)
C4—Te1—Br1	93.5 (2)	C16—C15—H15	119.9
Br2—Te1—Br1	90.55 (3)	C14—C15—H15	119.9
Br4—Te1—Br1	177.46 (3)	C15—C16—C11	118.9 (7)
C4—Te1—Br3	89.7 (2)	C15—C16—H16	120.5
Br2—Te1—Br3	176.86 (3)	C11—C16—H16	120.5
Br4—Te1—Br3	90.61 (3)	C22—C21—C26	119.2 (7)
Br1—Te1—Br3	87.11 (3)	C22—C21—P1	120.7 (6)
O1—P1—C21	112.0 (3)	C26—C21—P1	120.0 (6)
O1—P1—C11	104.1 (3)	C23—C22—C21	120.7 (7)
C21—P1—C11	110.7 (4)	C23—C22—H22	119.7
O1—P1—C31	107.9 (3)	C21—C22—H22	119.7
C21—P1—C31	110.2 (4)	C22—C23—C24	120.0 (8)
C11—P1—C31	111.8 (3)	C22—C23—H23	120.0
C1—O1—P1	121.6 (4)	C24—C23—H23	120.0
O1—C1—C2	107.2 (6)	C25—C24—C23	120.0 (7)
O1—C1—H1A	110.3	C25—C24—H24	120.0
C2—C1—H1A	110.3	C23—C24—H24	120.0
O1—C1—H1B	110.3	C24—C25—C26	120.9 (8)
C2—C1—H1B	110.3	C24—C25—H25	119.5
H1A—C1—H1B	108.5	C26—C25—H25	119.5
C1—C2—C3	115.3 (6)	C25—C26—C21	119.3 (8)
C1—C2—H2A	108.5	C25—C26—H26	120.4
C3—C2—H2A	108.5	C21—C26—H26	120.4
C1—C2—H2B	108.5	C36—C31—C32	119.7 (7)
C3—C2—H2B	108.5	C36—C31—P1	118.8 (6)
H2A—C2—H2B	107.5	C32—C31—P1	121.4 (6)
C4—C3—C2	110.0 (6)	C33—C32—C31	118.6 (8)
C4—C3—H3A	109.7	C33—C32—H32	120.7
C2—C3—H3A	109.7	C31—C32—H32	120.7
C4—C3—H3B	109.7	C34—C33—C32	120.6 (8)
C2—C3—H3B	109.7	C34—C33—H33	119.7
H3A—C3—H3B	108.2	C32—C33—H33	119.7
C3—C4—Te1	117.6 (5)	C35—C34—C33	120.3 (8)
C3—C4—H4A	107.9	C35—C34—H34	119.9
Te1—C4—H4A	107.9	C33—C34—H34	119.9
C3—C4—H4B	107.9	C34—C35—C36	119.5 (8)
Te1—C4—H4B	107.9	C34—C35—H35	120.3
H4A—C4—H4B	107.2	C36—C35—H35	120.3
C12—C11—C16	120.6 (7)	C31—C36—C35	121.3 (8)
C12—C11—P1	121.2 (6)	C31—C36—H36	119.4
C16—C11—P1	118.2 (6)	C35—C36—H36	119.4
C13—C12—C11	119.1 (7)	N1—C5—C6	178.8 (11)
C13—C12—H12	120.4	C5—C6—H6A	109.5
C11—C12—H12	120.4	C5—C6—H6B	109.5
C14—C13—C12	120.0 (8)	H6A—C6—H6B	109.5
C14—C13—H13	120.0	C5—C6—H6C	109.5
C12—C13—H13	120.0	H6A—C6—H6C	109.5
C13—C14—C15	121.1 (8)	H6B—C6—H6C	109.5
			
C21—P1—O1—C1	41.9 (6)	C31—P1—C21—C22	−148.6 (6)
C11—P1—O1—C1	161.5 (5)	O1—P1—C21—C26	−84.7 (6)
C31—P1—O1—C1	−79.6 (6)	C11—P1—C21—C26	159.7 (6)
P1—O1—C1—C2	−176.3 (5)	C31—P1—C21—C26	35.5 (7)
O1—C1—C2—C3	−67.3 (8)	C26—C21—C22—C23	−1.0 (11)
C1—C2—C3—C4	−67.7 (8)	P1—C21—C22—C23	−176.9 (6)
C2—C3—C4—Te1	−176.9 (5)	C21—C22—C23—C24	1.1 (12)
Br2—Te1—C4—C3	−62.1 (5)	C22—C23—C24—C25	−1.0 (11)
Br4—Te1—C4—C3	−153.9 (6)	C23—C24—C25—C26	0.9 (12)
Br1—Te1—C4—C3	28.3 (6)	C24—C25—C26—C21	−0.8 (12)
Br3—Te1—C4—C3	115.4 (5)	C22—C21—C26—C25	0.9 (11)
O1—P1—C11—C12	−11.4 (8)	P1—C21—C26—C25	176.8 (6)
C21—P1—C11—C12	109.2 (7)	O1—P1—C31—C36	−24.5 (7)
C31—P1—C11—C12	−127.6 (7)	C21—P1—C31—C36	−147.1 (6)
O1—P1—C11—C16	170.5 (6)	C11—P1—C31—C36	89.3 (7)
C21—P1—C11—C16	−69.0 (7)	O1—P1—C31—C32	158.1 (6)
C31—P1—C11—C16	54.2 (7)	C21—P1—C31—C32	35.5 (7)
C16—C11—C12—C13	0.1 (13)	C11—P1—C31—C32	−88.1 (7)
P1—C11—C12—C13	−178.0 (7)	C36—C31—C32—C33	0.9 (11)
C11—C12—C13—C14	−1.3 (14)	P1—C31—C32—C33	178.3 (6)
C12—C13—C14—C15	2.1 (14)	C31—C32—C33—C34	−1.3 (12)
C13—C14—C15—C16	−1.7 (13)	C32—C33—C34—C35	−0.1 (13)
C14—C15—C16—C11	0.5 (12)	C33—C34—C35—C36	2.1 (13)
C12—C11—C16—C15	0.3 (12)	C32—C31—C36—C35	1.1 (12)
P1—C11—C16—C15	178.5 (6)	P1—C31—C36—C35	−176.3 (6)
O1—P1—C21—C22	91.2 (7)	C34—C35—C36—C31	−2.6 (13)
C11—P1—C21—C22	−24.4 (7)		

### Hydrogen-bond geometry (Å, º)

**Table:**

*D*—H···*A*	*D*—H	H···*A*	*D*···*A*	*D*—H···*A*
C3—H3*B*···Br1	0.99	2.82	3.450 (7)	122
C26—H26···Br3^i^	0.95	2.75	3.619 (8)	152

Symmetry code: (i) −*x*+1, −*y*+1, −*z*.
